# Physical activity for psychological and physical well-being in menstruating individuals: an integrative review

**DOI:** 10.1186/s12905-026-04975-1

**Published:** 2026-10-06

**Authors:** Mareike Hechinger, Barbara Silva Salla, Milena Mühlbauer

**Affiliations:** Faculty of Applied Healthcare Sciences, Deggendorf Institute of Technology, Land-Au 25 + 27, Deggendorf, 94469 Germany

**Keywords:** Menstrual cycle, Women’s health, Well-being, Exercise, Menstrual mood changes, Physical activity, Primary dysmenorrhoea, Premenstrual syndrome, Quality of life

## Abstract

**Background:**

Physical and psychological symptoms vary across the menstrual cycle and may influence well-being and engagement in physical activity. While physical activity may alleviate menstrual pain, fatigue, and mood disturbances, evidence regarding beneficial types, intensities, and timing remains fragmented. This integrative review aimed to synthesise evidence on how physical activity contributes to psychological and physical well-being in menstruating individuals and to identify practically relevant recommendations.

**Methods:**

An integrative review was conducted using a PIO-based search strategy. CINAHL, PubMed/MEDLINE, and the Cochrane Library were searched for peer-reviewed studies published in English or German between 2010 and 2025, supplemented by backward and forward citation tracking. Qualitative, quantitative, and mixed-methods studies examining physical activity in relation to psychological and/or physical well-being across the menstrual cycle were eligible. Three reviewers independently screened records using predefined criteria. Methodological quality was assessed using the Mixed Methods Appraisal Tool (MMAT).

**Results:**

Fifty-five studies comprising 13,856 participants were included, with randomised controlled trials being most common (*n* = 31; 56.4%). Three domains emerged: menstrual pain and somatic symptoms; premenstrual and emotional symptoms; and quality of life, sleep, and daily functioning. Physical activity was predominantly associated with reductions in symptom burden and improvements in well-being. Evidence was most consistent for reductions in menstrual pain and somatic symptoms, particularly following mind–body approaches. Aerobic and other exercise modalities also showed beneficial but more heterogeneous findings. Improvements in premenstrual and emotional symptoms were frequently reported, whereas evidence for quality of life, sleep, and daily functioning was more limited. Heterogeneity in exercise modalities, intervention characteristics, and outcome assessment limited direct comparisons.

**Conclusions:**

Physical activity may improve psychological and physical well-being in menstruating individuals, with the most consistent evidence for menstrual pain and somatic symptoms; findings for premenstrual, emotional, and quality-of-life outcomes were less consistent. Mind–body approaches such as yoga and Pilates showed particularly promising results. Rather than supporting a uniform cycle-based prescription, the findings point towards an individualised and symptom-responsive approach that also takes underlying conditions into account. More standardised and methodologically robust research is needed to establish specific recommendations and better understand experiences of physical activity across the menstrual cycle.

**Supplementary Information:**

The online version contains supplementary material available at https://doi.org/10.1186/s12905-026-04975-1.

## Introduction

 Physical activity is a key conservative strategy for improving overall health. Consistent evidence indicates reductions in mortality, cardiometabolic risk, and symptoms of anxiety and depression, as well as benefits for sleep, energy, fatigue, and pain [1–3]. The World Health Organization (WHO) strongly recommends 150–300 min of moderate-intensity or 75–150 min of vigorous-intensity aerobic activity per week, alongside muscle-strengthening activities on at least two days per week [1].

Physical activity may be particularly relevant for menstruating individuals, as physical and psychological symptoms can vary across the menstrual cycle and may influence how physical activity is experienced and performed [4]. These variations are driven by cyclical hormonal fluctuations that are associated with a range of psychological and physical symptoms across the different phases of the menstrual cycle. Menstruating individuals commonly report premenstrual or menstrual complaints such as dysmenorrhoea, tiredness, and psychological symptoms [5, 6].

Symptoms such as fatigue, pain, and mood disturbances can act as barriers to physical activity and can restrict participation in school and work, with considerable interindividual variability in symptom patterns and severity [4, 6–8]. At the same time, many individuals report that physical activity can alleviate symptoms such as menstrual pain or low mood. However, it remains unclear which types, intensities, and timings of physical activity may be most beneficial [5].

Existing reviews have addressed distinct aspects of the relationship between the menstrual cycle and physical activity. One line of research has examined menstrual-cycle-related variation in exercise performance, focusing on how such changes can be managed or compensated for, rather than on well-being itself [9]. A second has examined the influence of menstrual symptoms on physical activity participation, rather than the reverse [10]. A third has evaluated the effectiveness of exercise for specific clinical outcomes, such as primary dysmenorrhoea, rather than physical and psychological well-being more broadly [11]. This fragmented evidence leaves scope for an integrative review that synthesises diverse study designs and perspectives to examine how physical activity may be used to support menstrual-cycle-related psychological and physical well-being.

This integrative review therefore aims to synthesise quantitative and qualitative evidence on how physical activity contributes to the psychological and physical well-being of menstruating individuals. It addresses the research question: ‘How does physical activity contribute to the psychological and physical well-being of menstruating individuals?’ The review further aims to identify evidence-based and practically feasible physical activity recommendations.

## Methods

### Study design

This integrative review adopted a systematic search strategy to identify and synthesise evidence from diverse methodological designs on how physical activity contributes to psychological and physical well-being in menstruating individuals. An integrative review approach was considered appropriate because it enables the inclusion and synthesis of evidence derived from diverse research methodologies [12, 13], thereby facilitating a more comprehensive understanding of how physical activity relates to psychological and physical well-being across the menstrual cycle. Relevant PRISMA 2020 reporting items [14] were considered to support transparent reporting of the search and study selection process.

### Eligibility criteria

Eligibility criteria were defined a priori and applied consistently throughout all stages of the screening process. Studies were eligible if they included menstruating individuals and examined physical activity in relation to psychological and/or physical well-being in the context of the menstrual cycle. This included studies that (a) examined the menstrual cycle as a whole, i.e., assessed outcomes at two or more cycle phases or repeatedly over at least one complete cycle; (b) focused on specific menstrual-cycle phases (e.g., menstruation or the luteal phase); or (c) addressed menstruation-related symptoms (e.g., menstrual pain or premenstrual symptoms). The population was intentionally defined broadly as menstruating individuals to capture the range of experiences and conditions relevant to menstrual-cycle-related well-being. In addition to individuals without a diagnosed menstrual-cycle-related condition, studies involving participants with primary dysmenorrhoea or premenstrual symptoms were eligible, as these symptoms are directly linked to specific cycle phases. Relevant experiences and outcomes included both subjective experiences (e.g., relating to well-being, quality of life, mood, pain, stress, sleep and daily functioning) and objective indicators of psychological and physical well-being. No restrictions were placed on the type of physical activity or comparator. Qualitative, quantitative, and mixed-methods studies were eligible for inclusion, reflecting the integrative nature of the review. Only peer-reviewed publications written in English or German and published between 2010 and 2025 were considered. Studies involving participants using hormonal contraception were excluded, as exogenous hormones suppress or alter the endogenous hormonal fluctuations that characterise the natural menstrual cycle. Animal studies, case reports, editorials, letters, study protocols without reported results, and studies that did not examine physical activity in relation to the menstrual cycle or specific cycle phases were also excluded.

### Information sources

The literature search was conducted in CINAHL, PubMed/MEDLINE, and the Cochrane Library in August 2025. The search in the Cochrane Library covered the Cochrane Database of Systematic Reviews (CDSR) and the Cochrane Central Register of Controlled Trials (CENTRAL). In addition, the reference lists of all included studies were screened, and forward citation tracking was undertaken to identify further relevant records.

### Search strategy

To structure the review and search strategy, a PIO framework (Population, Interest, Outcome) was applied. Outcome was deliberately understood to encompass both objectively measured outcomes and subjectively reported experiences, in keeping with the integrative nature of this review [15]. The research question was translated into three search components, corresponding to the PIO elements: (1) menstruating individuals, (2) physical activity, and (3) experiences or outcomes. Search terms for each component were derived from the corresponding MeSH terms and CINAHL Headings, combined with free-text terms, synonyms, truncation, and phrase searching where appropriate (see Supplementary Material 1 for the full search strategy). Electronic database searching was supplemented by backward reference screening of included studies and relevant reviews, and by forward citation tracking.

### Selection process and characteristics of included studies

All identified records were imported into Citavi (version 5), and duplicates were removed using automated detection followed by manual checking. The database search identified 3,465 records; after removal of duplicates (*n* = 509), 2,956 records remained for title and abstract screening. Screening was conducted using Rayyan review management software (Rayyan Systems Inc., Cambridge, MA 02142, United States) [16], and three reviewers independently applied the predefined eligibility criteria. Discrepancies were resolved through discussion between the reviewers and consensus. Of the screened records, 84 full-text articles were assessed for eligibility, and 55 studies met the inclusion criteria and were included in the review.

**Fig. 1:**
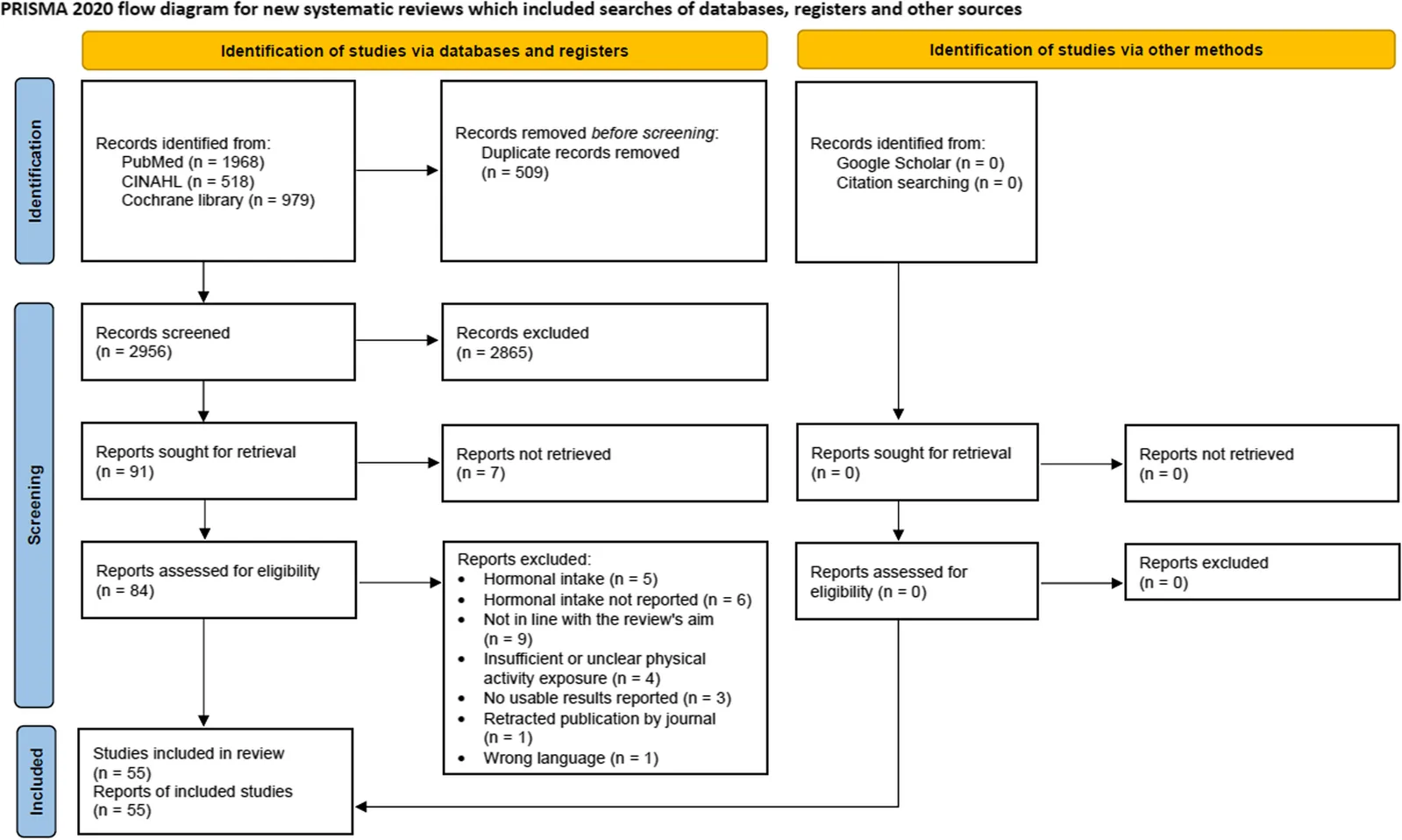
PRISMA flow diagram

The study selection process, including record identification, duplicate removal, title and abstract screening, full-text assessment, reasons for exclusion, and final study inclusion, is presented in the PRISMA flow diagram (Fig. 1) [14]. Reasons for exclusion at the full-text stage were documented and are provided in Supplementary Material 2. Data were extracted from the included studies by one reviewer and checked by a second reviewer. Any disagreements were resolved by discussion with a third reviewer. The main characteristics of the included studies, including study design, population, type of physical activity, and outcome measures, are summarised in Supplementary Material 3.

### Methodological quality assessment

Methodological quality was appraised using the Mixed Methods Appraisal Tool (MMAT) [17] (see Supplementary Material 4). The MMAT enables the critical appraisal of different study designs within a single framework and was therefore considered appropriate for this integrative review. Based on the study design, each study was assigned to the corresponding MMAT category and appraised using the respective design-specific criteria. Each criterion was rated as “Yes”, “No”, or “Can’t tell” in accordance with the MMAT guidance. No overall numerical quality score was calculated; instead, the individual criteria were considered to identify methodological strengths, limitations, and uncertainties across the included studies. The MMAT assessments were conducted independently by two reviewers. Any uncertainties or disagreements were discussed and resolved with a third reviewer. Detailed study-level assessments are provided in Supplementary Material 4.

Overall, the included studies were of variable quality. Common strengths included clearly stated aims, the use of validated instruments, and close alignment with the review question. Consistent with the data evaluation stage of the integrative review methodology, studies were included irrespective of methodological quality; quality appraisal instead informed the interpretation of findings [12].

## Results

### Study characteristics

A total of 55 studies, comprising 13,856 participants, were included in this integrative review. Sample sizes varied considerably across studies, ranging from 14 to 7,193 participants, with a median sample size of 65.

Across the included studies, the target population predominantly comprised young, otherwise healthy menstruating individuals, most commonly university or college students. Ages ranged from 12 to 80 years but concentrated within the 18–25-year range. One large survey-based study included women aged 16–80 years; among all 1,114 submitted responses, the mean age was 49 years and most participants were aged 45–59 years. Of these, 711 women reported menstrual symptoms and were included in the subsequent analyses; the age distribution of this subgroup was not reported separately.

The most frequently studied conditions were primary dysmenorrhoea (*n* = 17 studies) and premenstrual syndrome (PMS) or premenstrual symptoms (*n* = 11), while fewer studies addressed polycystic ovary syndrome (PCOS; *n* = 2), endometriosis (*n* = 1), or more general menstrual symptoms or difficulties (*n* = 4). Six studies included healthy or eumenorrhoeic participants without a specific menstrual-cycle-related condition. These categories were not mutually exclusive, as participants could meet more than one of these descriptions. Detailed information on the populations and conditions represented in each study is provided in Supplementary Material 3.

More than half of the included studies were randomised controlled trials (*n* = 31; 56.4%), followed by cross-sectional studies (*n* = 11; 20.0%). The remaining studies comprised pre–post studies (*n* = 4; 7.3%), randomised crossover trials (*n* = 2; 3.6%), quasi-experimental studies (*n* = 2; 3.6%), and non-randomised controlled, retrospective comparative, comparative, experimental, and exploratory within-subjects experimental studies (*n* = 1 each; 1.8%). No qualitative studies met the inclusion criteria.

Study location was reported for 54 of the 55 included studies, spanning 19 countries across five continents. The largest number of studies was conducted in Turkey (*n* = 11), Iran (*n* = 8), and India (*n* = 6), with the remaining studies distributed across other countries in Asia, Europe, the Middle East, North and South America, and Oceania.

The frequency of structured physical activity interventions ranged from once weekly to several sessions per day, with intervention periods ranging from 4 weeks to 12 months. Interventions varied in their mode of delivery, including professionally supervised or instructor-led sessions as well as programmes in which participants continued prescribed exercises independently after initial instruction. In addition, several studies examined habitual physical activity or existing training routines rather than implementing a structured exercise intervention.

Several studies evaluated multi-component programmes in which physical activity was combined with additional components, including education, positive psychology, breathing exercises, meditation, massage, acupressure, prayer, fasting, or dietary guidance. These programmes varied in the combination of components and in their mode of delivery.

Considerable heterogeneity was observed in outcome assessment across the included studies. Most studies predominantly relied on self-reported measures covering menstrual pain and somatic symptoms, premenstrual symptoms, psychological and emotional well-being, quality of life, physical activity, and sleep. Pain was commonly assessed using the Visual Analogue Scale (VAS) or numerical rating scales, while menstrual and premenstrual symptoms were frequently evaluated using instruments such as the Premenstrual Syndrome Scale (PMSS), Menstrual Distress Questionnaire (MDQ), Menstrual Symptoms Questionnaire (MSQ), and Premenstrual Symptoms Screening Tool (PSST). Psychological outcomes were assessed using measures including the Depression Anxiety Stress Scale (DASS), Beck inventories, Perceived Stress Scale (PSS), and Profile of Mood States (POMS), whereas quality of life was commonly evaluated using the Short Form Health Survey (SF-36/SF-12) or World Health Organization Quality of Life (WHOQOL) instruments. Physical activity was frequently assessed using the International Physical Activity Questionnaire (IPAQ, long or short form). Objective measures, including accelerometry, hormone levels, pressure pain thresholds, and heart rate variability, were used less frequently.

### Main findings

Consistent with the integrative review framework proposed by Whittemore and Knafl (2005), extracted data were iteratively compared and reduced to identify recurring patterns across studies. This analytic process resulted in three domains describing the relationship between physical activity and menstrual-cycle-related well-being: (1) menstrual pain and somatic symptoms, (2) premenstrual and emotional symptoms, and (3) quality of life, sleep and daily functioning. The allocation of individual studies to each domain is provided in Supplementary Material 3. An overview of the evidence synthesised across the three domains is provided in Table 1.

**Table 1:** Summary of findings

Outcome domain	Evidence base	Summary of findings
Menstrual pain and somatic symptoms	43 studies; RCTs, observational studies, and other quantitative designs	Reductions in menstrual pain and somatic symptom burden were predominantly reported in association with physical activity. Mind–body approaches, including yoga, Pilates and Baduanjin, showed particularly consistent beneficial findings, while aerobic and other exercise programmes produced more heterogeneous results.
Premenstrual and emotional symptoms	37 studies; RCTs, observational studies, and other quantitative designs	Structured physical activity was predominantly associated with improvements in premenstrual and emotional symptoms. Findings were generally favourable across aerobic and mind–body approaches, although effects were more heterogeneous for individual psychological outcomes such as anxiety and depression.
Quality of life, sleep and daily functioning	26 studies; RCTs, observational studies, and other quantitative designs	Improvements were reported in quality of life, sleep, and daily functioning, including physical functioning, general health perception, concentration, participation in school or work, and sleep quality. However, findings were not consistent across all outcomes, with some studies reporting no significant effects or unfavourable associations.

Across these domains, the evidence was most consistent for reductions in menstrual pain and related somatic symptoms, particularly following mind-body exercise approaches, whereas findings for psychological and quality-of-life outcomes were more heterogeneous and the evidence base for the latter remained comparatively limited.

The type and delivery of physical activity varied considerably across the included studies. The following classification reflects the movement-based component of each study; non-movement elements of multi-component programmes (e.g., informational sessions used as control conditions) were not considered. Intervention studies most frequently investigated yoga or yoga-based exercise [18–31], followed by aerobic exercise, including walking, treadmill training, aerobic dance, swimming, and aquatic exercise [32–42]. Other approaches included Pilates [43–45], stretching [32, 45–49], strengthening or resistance exercise [48, 50], high-intensity interval training (HIIT) [51–53], Baduanjin [54, 55], and combined exercise programmes incorporating multiple movement components such as aerobic, strengthening, stretching, mobility, and breathing exercises [56–60]. Several studies did not implement a standardised exercise intervention but examined habitual physical activity, existing training routines, or differences between physically active and less active or sedentary participants [61–72].

#### Domain 1: menstrual pain and somatic symptoms

Across the included literature, 43 of the 55 included studies examined the relationship between physical activity and menstrual pain or related somatic symptoms, most commonly among individuals experiencing primary dysmenorrhoea or other menstrual-related pain conditions.

Across these intervention studies, mind-body exercise approaches such as yoga, Pilates, and Baduanjin were frequently associated with reductions in menstrual pain intensity and menstrual distress scores [18, 19, 22–29, 31, 32, 36–38, 43–45, 47, 49, 50, 54–60, 70, 71]. Observational studies similarly indicated that individuals with higher levels of habitual physical activity reported lower levels of menstrual pain or somatic symptom burden [62, 63, 65, 68, 70, 71]. One observational study found the opposite pattern: athletes with a higher training frequency reported a higher, not lower, prevalence of PMS, suggesting that more habitual physical activity does not always relate to fewer symptoms [61]. Several studies reported significant improvements in pain severity measured using VAS or MSQ following structured exercise programmes [18, 20, 22, 24, 28, 29, 32–34, 36–38, 45–48, 50, 57–60, 62, 63, 65, 66].

Aerobic exercise interventions and other exercise programmes were also associated with beneficial effects on menstrual pain, although findings were somewhat more heterogeneous across studies [24, 25, 28, 32–34, 36, 39, 45, 46, 48, 54]. In addition, a physiotherapy-based exercise programme was linked to improvements in abdominal pain, menstrual-related low back pain, and other somatic symptoms [59].

Beyond pain, structured exercise programmes were associated with reductions in a range of other somatic symptoms, including headache, breast tenderness, abdominal bloating or swelling, fatigue, and menstrual cramps [22, 23, 27, 39, 44, 59, 68, 71].

However, findings were not entirely consistent across all studies. Some investigations reported only within-group improvements or no statistically significant association between habitual physical activity levels and pain severity [37, 64, 66]. Differences in intervention duration, exercise intensity, and outcome measurement may partly explain these inconsistencies. Overall, the available evidence suggests that structured physical activity may contribute to reductions in menstrual pain and somatic symptom burden, although exercise modalities varied across included studies.

#### Domain 2: premenstrual and emotional symptoms

37 of the 55 included studies investigated the relationship between physical activity and premenstrual symptoms as well as emotional well-being, including outcomes such as mood disturbances, irritability, stress, anxiety, and depressive symptoms.

Across intervention studies, exercise programmes such as yoga, aerobic exercise, and mind-body training were associated with improvements in emotional and psychological symptoms related to the premenstrual phase, with these outcomes often assessed using validated instruments such as the PMSS, MDQ, or other psychological symptom scales.

Participants engaging in regular exercise reported reductions in irritability, mood swings, and psychological distress in several studies [19–21, 23, 26, 28, 30–32, 35, 39, 40, 42, 44, 46, 48–51, 53–57, 67, 72]. Observational studies similarly indicated that individuals with higher levels of habitual physical activity reported lower levels of stress, depressive symptoms, or premenstrual symptom burden [62, 64, 65, 68, 69, 71]. In contrast, athletes with higher training frequency reported more frequent mood lability, irritability, and anger than non-athlete controls in one observational study [61].

One study found that improvements in menstrual distress and stress following an 8-week yoga programme were partially maintained at 24 weeks, though effects diminished as most participants discontinued practice [20]. One study similarly found that engagement in exercise was associated with improved affective valence and motivation during the session across all cycle phases, despite pre-exercise anxiety and negative affect being higher during menstruation than in other phases [35]. One further study found that psychological responses to exercise, such as mood and affect, were more negative during the luteal phase than during the follicular phase [41].

However, findings varied across studies, and not all interventions produced significant improvements in psychological outcomes. Variations in exercise modality, intervention duration, and measurement instruments may partly account for the heterogeneity of the results. One study found a significant reduction in premenstrual symptoms and pain following Pilates, but an unexpected increase in perceived stress in the intervention group [43]. Some investigations reported no significant improvement in psychological outcomes such as anxiety or depression despite improvements in physical symptoms [34].

One study additionally found that stress levels were associated with the perceived intensity of menstrual pain [70]. Overall, the findings suggest that physical activity may have beneficial effects on emotional and premenstrual symptoms, although the strength and consistency of these effects vary across studies.

#### Domain 3: quality of life, sleep and daily functioning

Across the included literature, 26 of the 55 included studies examined the relationship between physical activity and broader indicators of quality of life, sleep, and daily functioning. These outcomes were typically measured using instruments such as the Short Form Health Survey (SF-36) or other quality-of-life questionnaires.

Across these studies, participation in structured exercise programmes was associated with improvements in several domains of quality of life, including physical functioning, general health perception, and psychological well-being, while some studies also reported reduced disruption of daily activities during menstruation, such as improved concentration, lower absenteeism, and better participation in school or work activities [18, 20, 21, 24, 27, 29, 31, 33, 34, 38, 45–48, 52, 53, 57, 58, 72]. One observational study found that sedentary women were more likely to report that menstrual symptoms interfered with their social, personal, and family life than physically active women [68]. In contrast, one observational study found that athletes with higher training loads reported more concentration difficulties, absenteeism, and poorer academic performance [61]. One study found no significant effect of habitual physical activity intensity on menstruation-related quality of life [64].

A smaller number of studies also assessed sleep quality, with structured exercise interventions associated with improvements in sleep quality in some [48, 54, 57], though not all, studies [38]. One further study reported a reduction in daytime sleepiness following the intervention [58]. Sleep-related complaints, such as insomnia, fatigue or sleep disturbances, were also captured as individual items within broader premenstrual symptom checklists in several further studies. Most of these studies reported improvements in sleep-related items following exercise [23, 39, 44], although one study found no clear change [21]. As these items were embedded within composite symptom scores rather than assessed as stand-alone outcomes, study-specific effects on sleep could not be reliably isolated.

Nevertheless, the evidence base for quality-of-life outcomes was more limited compared with research focusing on pain or symptom severity. In addition, methodological differences across studies make direct comparisons challenging. Overall, the available findings suggest that physical activity may contribute to improvements in perceived well-being, sleep and daily functioning, although further research is needed to strengthen the evidence base in this area.

## Discussion

This integrative review synthesised evidence from 55 studies examining the relationship between physical activity and psychological and physical well-being across the menstrual cycle. Overall, physical activity was most consistently associated with reductions in menstrual pain and somatic symptom burden. Improvements in premenstrual and emotional symptoms, such as reduced irritability, mood disturbances, and psychological distress, were also observed, although less consistently. Improvements in quality of life, sleep, and daily functioning, such as better concentration and participation in school or work, were also reported in most studies, although the evidence base for this domain was smaller and less consistent than for pain-related outcomes, with a small number of studies reporting no clear effect, unfavourable associations with higher physical activity levels, or no change in sleep-related outcomes. However, substantial heterogeneity across studies limits conclusions regarding the optimal type, intensity, duration, and timing of physical activity.

### Effects of physical activity on menstrual health

The review suggests that structured physical activity may constitute a relevant non-pharmacological approach to supporting menstrual health, with the clearest benefits observed for dysmenorrhoea and related somatic symptoms. Mind–body approaches such as yoga, Pilates, and Baduanjin showed particularly favourable findings across several studies, while findings for aerobic and other exercise modalities were more variable.

This pattern is broadly consistent with prior meta-analytic evidence: a systematic review and meta-analysis of self-care interventions for primary dysmenorrhoea found that exercise produced the largest effect size for pain reduction among non-pharmacological approaches, outperforming heat therapy and acupressure [73]. Similarly, a meta-analysis restricted to physical activity interventions for primary dysmenorrhoea reported moderate-quality evidence for reductions in pain intensity [74], and a further meta-analysis of yoga-based interventions for premenstrual syndrome reported reductions in menstrual distress alongside physiological benefits [75]. These findings support the present review’s observation that mind-body and structured exercise interventions represent a comparatively well-evidenced non-pharmacological approach, although effect sizes and study quality varied across the underlying meta-analyses.

### Adapting physical activity to menstrual-cycle-related symptoms

The findings also raise the possibility that adapting physical activity to cycle-related changes may be relevant for menstrual-cycle-related well-being. Consistent with prior evidence indicating that menstrual symptoms shape engagement in physical activity [10], our findings suggest that physical activity may, in turn, alleviate pain, distress, and functional limitations – pointing to a potentially bidirectional relationship worth exploring in future research. One potential explanation for this relationship may involve interactions between premenstrual symptomatology and stress reactivity: emerging evidence suggests that PMS and stress-response systems share overlapping hormonal circuits, and that PMS itself may act as a stressor, potentially reinforcing a cycle in which symptom burden further reduces motivation and capacity for physical activity [76]. In this regard, interventions that combine movement with guidance, self-observation, and symptom-responsive adaptation may be particularly beneficial, as suggested by the positive findings for yoga-based, mindfulness-based, and combined educational approaches [20, 26, 30, 42, 44, 56, 60, 72].

### Heterogeneity of physical activity

A further consideration is that the heterogeneity of findings may partly reflect the broad and multidimensional nature of physical activity and the variability in how it was operationalised across studies. Across studies, this ranged from structured mind-body approaches to habitual or self-reported activity levels. This diversity limits direct comparability and makes it difficult to determine which forms of movement are most relevant for menstrual-cycle-related well-being. One possible explanation for these findings is that mind-body interventions combine physical activity with components such as relaxation, breath regulation, body awareness, and stress reduction [18, 20, 22, 24–31, 60]. In contrast, more conventional exercise interventions may differ more substantially in intensity, supervision and adherence, which may partly explain the greater variability in findings [33, 34, 36–39, 51–53].

This challenge may be particularly relevant in the context of the menstrual cycle, where experiences of symptoms and their impact on physical activity vary substantially between individuals. A systematic review of recreational physical activity reported considerable variability in menstrual symptoms, experiences, and perceptions and consequently highlighted the need for an individualised approach to physical activity across the menstrual cycle [4].

Qualitative findings similarly suggest that women differ in whether and how they adapt their activity according to how they feel during different phases, with some preferring lighter activities, others continuing their usual routines, and some avoiding activity altogether due to concerns such as discomfort [77]. This variability points toward the potential value of combined, individualised approaches: meta-analytic evidence suggests that combined self-management strategies, including physical activity alongside nutritional and behavioural approaches, may be more effective in alleviating menstrual-cycle symptoms than physical activity alone [78]. Thus, heterogeneity in the type and delivery of physical activity may not merely represent methodological inconsistency but may also reflect meaningful differences in how physical activity is selected, experienced, and adapted across the menstrual cycle.

### Limitations and strengths

Several limitations of this review should be acknowledged. The included studies displayed substantial heterogeneity in study design, population characteristics, intervention content, and outcome measurement, a common characteristic of integrative reviews. This heterogeneity extended to the conditions studied, including primary dysmenorrhoea, premenstrual symptoms, endometriosis, and polycystic ovary syndrome. In addition, a number of studies evaluated multi-component interventions in which physical activity was combined with additional components, such as education, psychological approaches, breathing exercises, meditation, massage, acupressure, prayer, fasting, or dietary guidance. In these studies, the observed effects cannot be attributed to physical activity alone.

Consistent with the narrative synthesis approach of integrative reviews, no pooled effect sizes were calculated, so the magnitude of associations could not be quantitatively compared across studies. The concept of physical activity itself was broadly operationalised, limiting differentiation between types, intensities, and durations and making it difficult to determine which characteristics may be most beneficial. Outcomes relating to psychological well-being, quality of life, sleep, and daily functioning were investigated less frequently than pain-related and somatic outcomes, resulting in a comparatively less robust evidence base in these domains.

Most studies relied on self-reported rather than objective measures of symptoms and physical activity, increasing susceptibility to recall and reporting bias, and methodological quality varied across studies, so findings should be interpreted with these limitations in mind. The inclusion of both intervention and observational designs enabled a broad synthesis but limits causal interpretation, as associations identified in observational studies cannot establish whether physical activity itself caused the observed differences.

Although qualitative studies were eligible, none met the inclusion criteria; the synthesis therefore predominantly reflects quantitative evidence and may not fully capture lived experiences, individual perceptions, and contextual factors. Finally, the search was restricted to peer-reviewed publications in English or German (2010–2025) across three databases (CINAHL, PubMed/MEDLINE, and the Cochrane Library); despite backward and forward citation tracking, relevant evidence in other languages, outside this period, in grey literature, or indexed elsewhere may have been missed. A further limitation is that the literature search was conducted in August 2025 and was not updated before submission. However, the included studies showed a consistent overall pattern of findings across the three outcome domains.

At the same time, the review has notable strengths. Its integrative approach conceptualises menstrual-cycle-related well-being as multidimensional rather than reducing it to pain alone, and the inclusion of diverse methodological designs enabled synthesis of both intervention effects and observational associations, capturing physical, psychological, functional, and behavioural dimensions of physical activity. Methodological quality was systematically appraised using design-specific MMAT criteria, allowing findings to be interpreted considering the strengths and limitations of the underlying evidence.

## Conclusions

This integrative review indicates that physical activity may contribute to improved psychological and physical well-being in menstruating individuals, with the most consistent evidence relating to reductions in menstrual pain and somatic symptoms. Findings for premenstrual and emotional symptoms, quality of life, sleep, and daily functioning were generally favourable but less consistent. Mind-body approaches such as yoga and Pilates showed particularly promising results, although the substantial heterogeneity in physical activity modalities, populations, and outcome measures precludes conclusions about the optimal type, intensity, duration, or timing of physical activity.

Overall, the findings support physical activity as a potentially relevant non-pharmacological approach to menstrual-cycle-related well-being. Given the substantial variability in symptoms and individual responses across the menstrual cycle, an individualised and symptom-responsive approach may be a relevant consideration for clinical practice and future research. Such an approach should consider personal experiences, preferences, underlying conditions, and changes in well-being across the menstrual cycle. More standardised and methodologically robust research is needed to establish specific physical activity recommendations and to better understand psychological, functional, and lived experiences alongside physical symptoms.

Future research should apply more standardised methodological approaches, use consistent definitions and assessment methods for menstrual-cycle phases, define and report physical activity more precisely, and incorporate objective measures alongside self-reported outcomes. Greater attention should also be given to psychological well-being, quality of life, sleep, daily functioning, lived experience, and adaptive self-management. Given that the included studies predominantly focused on younger, otherwise healthy menstruating individuals, future research should examine how the relationship between physical activity and menstrual-cycle-related well-being may vary across age groups and different stages of the reproductive life course, including the years approaching menopause and among individuals with different reproductive histories. Future studies should also examine potential moderators and mediators of the relationship between physical activity and menstrual-cycle-related well-being, including menstrual-cycle phase, stress, and the level and frequency of physical activity, to better understand for whom, under which conditions, and through which mechanisms physical activity may influence symptoms and well-being. Qualitative and mixed-methods research may be particularly valuable for understanding how menstruating individuals perceive and adapt physical activity in response to cycle-related symptoms and changes in well-being.

## Supplementary Information

Supplementary Material 1.

Supplementary Material 2.

Supplementary Material 3.

Supplementary Material 4.

## Data Availability

All data supporting the findings of this study are available within the paper and its Supplementary Information.
