# Supplementary material for: Physical activity for psychological and physical well-being in menstruating individuals: an integrative review
Source: BMC Womens Health. 2026 Oct 6;26:502. doi: 10.1186/s12905-026-04975-1 (PMC13640090; doi:10.1186/s12905-026-04975-1)
Supplement: Supplementary file 1 [file 12905_2026_4975_MOESM1_ESM.docx]

|  | **PubMed** | **Results (n)** | **CINAHL** | **Results (n)** | **Cochrane library** | **Results (n)** |
| --- | --- | --- | --- | --- | --- | --- |
| **Search  component 1:  Menstruating Individuals** | Menstruation [MesH]  Menstrual cycle [MesH]  Menstru*[Title/ Abstract]  “Follicular phase” [Title/ Abstract]  “Luteal phase” [Title/ Abstract]  Ovulation [Title/ Abstract]  Premenstru* [Title/ Abstract]  PMS [Title/ Abstract]  Menstruation disturbances [MeSH]  “Menstruation disturbances” [Title/ Abstract]  “fertile period” [Title/ Abstract] | 138 555 | MH Menstruation OR MH menstrual cycle OR XB menstru* OR XB "follicular phase" OR XB "luteal phase" OR XB ovulation OR XB premenstru* OR XB PMS OR MH Menstruation disorders OR XB "menstruation disturbances" OR XB "fertile period" | 20 861 | Menstruation [MesH]  Menstrual cycle [MesH]  Menstru*  “Follicular phase” [Title/ Abstract]  “Luteal phase” [Title/ Abstract]  Ovulation [Title/ Abstract]  Premenstru* [Title/ Abstract]  PMS [Title/ Abstract]  Menstruation disturbances [MeSH]  “Menstruation disturbances” [Title/ Abstract]  “fertile period” [Title/ Abstract] | 23 484 |
| **Search  component 2:  Physical Activity** | Movement [MeSH]  Movement [Title/ Abstract]  “Physical activit* “[Title/ Abstract]  “Physical exercise” [Title/ Abstract]  Exercise [MesH]  Exercise therapy [MesH]  Exercise movement techniques [MesH]  Activit* [Title/ Abstract]  Training [Title/ Abstract]  Mobili* [Title/ Abstract]  Sports [MesH]  Sport* [Title/ Abstract] | 5 573 439 | MH movement OR XB movement OR XB "physical activit*" OR XB "physical exercise" OR MH physical activity OR MH exercise OR XB “Exercise therapy“ OR XB “Exercise movement techniques” OR XB Activit* OR XB training OR XB mobilit* OR sports OR MH sports | 864 669 | Movement [MeSH]  Movement [Title/ Abstract]  “physical activities”  “physical activity”  Exercise [MesH]  Exercise therapy [MesH]  Exercise movement techniques [MesH]  Activit* [Title/ Abstract]  Training [Title/ Abstract]  Mobili* [Title/ Abstract]  Sports [MesH]  Sport* [Title/ Abstract] | 421 508 |
| **Search  component 3:   Experiences or outcomes** | Psychological well-being [MesH]  “well-being” [Title/ Abstract]  Physical well-being [Title/ Abstract]  Mood [Title/ Abstract]  Personal satisfaction [MesH]  Quality of Life [MesH]  “quality of life” [Title/ Abstract]  Emotions [MesH]  Pain [MesH]  Emotional regulation [MesH]  Sleep [MeSH]  Sleep [Title/ Abstract]  Stress, psychological [MeSH]  Stress [Title/ Abstract]  Resilience, psychological [MeSH]  Resilience [Title/ Abstract] | 3 609 911 | MH psychological well-being OR XB "well-being" OR XB "physical well-being" OR XB mood OR MH personal satisfaction OR MH quality of life OR XB "Quality of life" OR MH emotions OR MH pain OR MH emotional regulation OR MH sleep OR XB sleep OR MH stress OR XB stress OR XB resilience | 717 526 | Psychological well-being [MesH]  “well-being” [Title/ Abstract]  “Physical well-being” [Title/ Abstract]  Mood [Title/ Abstract]  Personal satisfaction [MesH]  Quality of Life [MesH]  “quality of life” [Title/ Abstract]  Emotions [MesH]  Pain [MesH]  Emotional regulation [MesH]  Sleep [MeSH]  Sleep [Title/ Abstract]  Stress, psychological [MeSH]  Stress [Title/ Abstract]  Resilience, psychological [MeSH]  Resilience [Title/ Abstract] | 405 798 |
| Search | 1 AND 2 AND 3 – 2010 to 7 August 2025 | 1968 | 1 AND 2 AND 3 – 2010 to 7 August 2025 | 518 | 1 AND 2 AND 3 – 2010 to 8 August 2025 | 979 |
| Search string | ( "Menstruation"[MeSH] OR "Menstrual Cycle"[MeSH] OR menstru*[Title/Abstract] OR "follicular phase"[Title/Abstract] OR "luteal phase"[Title/Abstract] OR ovulation[Title/Abstract] OR premenstru*[Title/Abstract] OR PMS[Title/Abstract] OR "Menstruation Disturbances"[MeSH] OR "menstruation disturbances"[Title/Abstract] OR "fertile period"[Title/Abstract] ) AND ( "Movement"[MeSH] OR movement[Title/Abstract] OR "physical activit*"[Title/Abstract] OR "physical exercise"[Title/Abstract] OR "Exercise"[MeSH] OR "Exercise Therapy"[MeSH] OR "Exercise Movement Techniques"[MeSH] OR activit*[Title/Abstract] OR training[Title/Abstract] OR mobili*[Title/Abstract] OR "Sports"[MeSH] OR sport*[Title/Abstract] ) AND ( "Psychological Well-Being"[MeSH] OR "well-being"[Title/Abstract] OR "physical well-being"[Title/Abstract] OR mood[Title/Abstract] OR "Personal Satisfaction"[MeSH] OR "Quality of Life"[MeSH] OR "quality of life"[Title/Abstract] OR "Emotions"[MeSH] OR "Pain"[MeSH] OR "Emotional Regulation"[MeSH] OR "Sleep"[MeSH] OR sleep[Title/Abstract] OR "Stress, Psychological"[MeSH] OR stress[Title/Abstract] OR "Resilience, Psychological"[MeSH] OR resilience[Title/Abstract] ) AND ("2010/01/01"[Date - Publication] : "2025/08/07"[Date - Publication]) |  | ( MH Menstruation OR MH "Menstrual Cycle" OR XB menstru* OR XB "follicular phase" OR XB "luteal phase" OR XB ovulation OR XB premenstru* OR XB PMS OR MH "Menstruation Disorders" OR XB "menstruation disturbances" OR XB "fertile period" ) AND ( MH Movement OR XB movement OR XB "physical activit*" OR XB "physical exercise" OR MH "Exercise" OR MH "Exercise Therapy" OR MH "Exercise Movement Techniques" OR XB activit* OR XB training OR XB mobilit* OR MH Sports OR XB sport* ) AND ( MH "Psychological Well-Being" OR XB "well-being" OR XB "physical well-being" OR XB mood OR MH "Personal Satisfaction" OR MH "Quality of Life" OR XB "quality of life" OR MH Emotions OR MH Pain OR MH "Emotional Regulation" OR MH Sleep OR XB sleep OR MH "Stress, Psychological" OR XB stress OR MH "Resilience, Psychological" OR XB resilience ) AND DT 20100101-20250807 |  | (MeSH descriptor: [Menstruation] explode all trees OR MeSH descriptor: [Menstrual Cycle] explode all trees OR (Menstru*):ti,ab,kw OR ("follicular phase"):ti,ab,kw OR ("luteal phase"):ti,ab,kw OR (ovulation):ti,ab,kw OR (premenstru*):ti,ab,kw OR (PMS):ti,ab,kw OR MeSH descriptor: [Menstruation Disturbances] explode all trees OR ("menstruation disturbances"):ti,ab,kw OR ("fertile periode"):ti,ab,kw) AND (MeSH descriptor: [Movement] explode all trees OR (Movement):ti,ab,kw OR ("physical activities"):ti,ab,kw OR ("physical activity"):ti,ab,kw OR MeSH descriptor: [Exercise] explode all trees OR MeSH descriptor: [Exercise Therapy] explode all trees OR MeSH descriptor: [Exercise Movement Techniques] explode all trees OR (activit*):ti,ab,kw OR (training):ti,ab,kw OR (mobili*):ti,ab,kw OR (sport*):ti,ab,kw OR MeSH descriptor: [Sports] explode all trees) AND (MeSH descriptor: [Psychological Well-Being] explode all trees OR (well-being):ti,ab,kw OR ("physical well-being"):ti,ab,kw OR (mood):ti,ab,kw OR MeSH descriptor: [Personal Satisfaction] explode all trees OR MeSH descriptor: [Quality of Life] explode all trees OR ("quality of life"):ti,ab,kw OR MeSH descriptor: [Emotions] explode all trees OR MeSH descriptor: [Pain] explode all trees OR MeSH descriptor: [Emotional Regulation] explode all trees OR MeSH descriptor: [Sleep] explode all trees OR (sleep):ti,ab,kw OR MeSH descriptor: [Stress, Psychological] explode all trees OR (stress):ti,ab,kw OR MeSH descriptor: [Resilience, Psychological] explode all trees OR (resilience):ti,ab,kw) |  |
